# Diagnostic Problem in a Child With an Isolated Renal Hydatid Cyst: A Case Report

**DOI:** 10.7759/cureus.60319

**Published:** 2024-05-15

**Authors:** Valeri Velev, Teofil Pelov, Gabriela Minova, Petar-Preslav Petrov, Stoyan Peev, Edmond Rangelov, Plamen Penchev

**Affiliations:** 1 Department of Infectious Diseases and Parasitology, University Hospital “Prof. Iv. Kirov”, Sofia, BGR; 2 Department of Infectious Diseases and Parasitology, Medical University Sofia, Sofia, BGR; 3 Department of Pediatric Urology, University Hospital “N. I. Pirogov”, Sofia, BGR; 4 Department of Anatomy, Histology and Embryology, Medical University of Plovdiv, Plovdiv, BGR; 5 Department of Pediatric Surgery, University Hospital “N. I. Pirogov”, Sofia, BGR; 6 Faculty of Medicine, Medical University of Plovdiv, Plovdiv, BGR

**Keywords:** ct examination, case report, hematuria, renal hydatid cyst, hydatid disease

## Abstract

Cystic hydatid disease is a parasitic disease caused by the larvae of the small tapeworm *Echinococcus granulosus*. It is still a serious public health problem in endemic regions such as the Mediterranean basin, especially in the Balkans. Usually, the complaints caused by the cysts are non-specific and there are rarely abnormalities in routine laboratory tests. The most common is the involvement of the liver. The frequency of isolated kidney involvement, especially in a child, is uncommon. We describe a rare pediatric case of an isolated renal hydatid cyst presenting with a urinary tract infection-like clinical presentation, leading to misdiagnosis and delayed treatment.

## Introduction

Cystic echinococcosis is a chronic relapsing parasitic disease caused by a small nematode Echinococcus granulosus. The disease is a typical endemic zoonosis, with areas of high incidence most often associated with well-developed sheep farming. In Europe, these areas are the Mediterranean, the Balkans, areas of South-Eastern Europe, and in the world, South America, New Zealand, parts of Africa, and the Middle East. In 2021, for EU/EEA the highest notification rates were observed in Bulgaria (1.27 cases per 100,000 population) [1,2]. The final host, and source of infection, is mainly a family of dogs, most often infected by eating parasitized guts of herbivores, which are intermediate hosts. A human accidentally enters the chain as an intermediate host by ingesting infective taenia eggs. The larval forms (hydatid cysts) in the intermediate host, including humans; they can get into any tissue or organ, but they are most often localized in the liver (over 75%).

Isolated renal echinococcosis, especially in children, is extremely rare (1-1.9%) in the world. In Bulgaria, the frequency is described between 1.3 and 3.4 % [2] and its diagnosis is a serious challenge, especially in the early asymptomatic stages [1-3]. In very rare cases, non-specific symptoms such as heaviness or pain in the lumbar region, fatigue, nausea, and less commonly hematuria and the presence of hydatid vesicles in the urine appear. Diagnostic tools are ultrasound studies, contrast-enhanced CT, and serological tests. Treatment protocols for renal echinococcosis are not established, most authors recommend open surgery [3,4]. The aim of this case is to show the diagnostic difficulties in this rare diagnosis and the ways to manage the situation.

## Case presentation

A 13-year-old boy presented to his general practitioner (GP) with a moderately elevated temperature, dysuria, "dark urine" and intermittent pain in the right half of the abdomen and lower back. During the physical examination, the doctor did find only palpable pain in the right lumbar region. From the appointed complete blood count, there was only moderate leukocytosis (15.6; normal ranges 4.00-11.00 x109/L) with elevated CRP (26.0; normal ranges 8-10 mg/L) and discrete anemia (hemoglobin 86; normal ranges 120-180 g/L). A simple urinalysis showed 12-15 leukocytes per microscopic field and single erythrocytes (5-6 per microscopic field) in the sediment. The rest of the parameters were normal. The doctor made a working diagnosis of urinary tract infection and started empiric therapy with trimethoprim/sulfamethoxazole 40mg/200mg two tablets daily for 10 days. After starting the treatment, the child subjectively felt better, the temperature decreased on the second day, but the pain and dysuria continued. On the fourth day, the child's temperature rose again to 38.0 °C, and the urine became "reddish." After another visit to the GP, only urine was examined for the child, 7-8 leukocytes were observed per microscopic field, and erythrocytes were 15- 20 per field.

Due to the "lack of significant changes in the urine", the parent was advised to continue the child's treatment with the already-mentioned medication. In the evening of the fifth day, the pain increased to the point of intolerance, and the temperature persisted. The parents were referred to a pediatric urology clinic. While waiting in front of the emergency room, the child wanted to urinate and the emergency physician noticed macroscopic hematuria. A little later, the child collapsed briefly in front of the office.

On physical examination, he was hemodynamically stable, but with tachycardia, and the patient reacted with pain in the area of ​​the right kidney. From the laboratory blood tests, no more serious than the described inflammatory changes were observed, there was no eosinophilia, and the renal biochemistry (urea and creatinine) was normal. Single leukocytes and epithelial cells with numerous erythrocytes were observed in the urine sediment. Abdominal US revealed a thin-walled echogenic cystic lesion at the upper pole of the right kidney measuring 90 mm in diameter.

**Figure 1:**
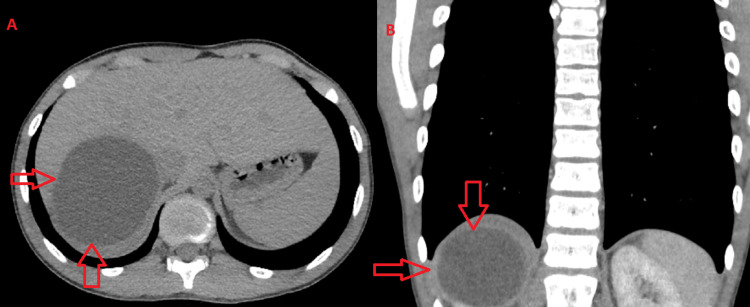
Preoperative abdominal CT scan A: CT scan; axial plane. B: CT scan; coronal plane

Contrast-enhanced CT of the upper pole of the right kidney showed a large cystic lesion with dense outer walls with marked enhancement along the course of the post-contrast series. The described finding has dimensions of 76x88x97 mm, respectively in the axillary/sagittal/coronary plane, compressing and pushing the right liver lobe ventrally. There are no solid segments or septa in the cyst (Figure 1). A minimal amount of fluid was observed around the dorsal contour of the cyst. Compression was present over the right adrenal gland, which was displaced medially. The parenchyma in the middle third and the lower pole of the kidney was preserved.

**Figure 2:**
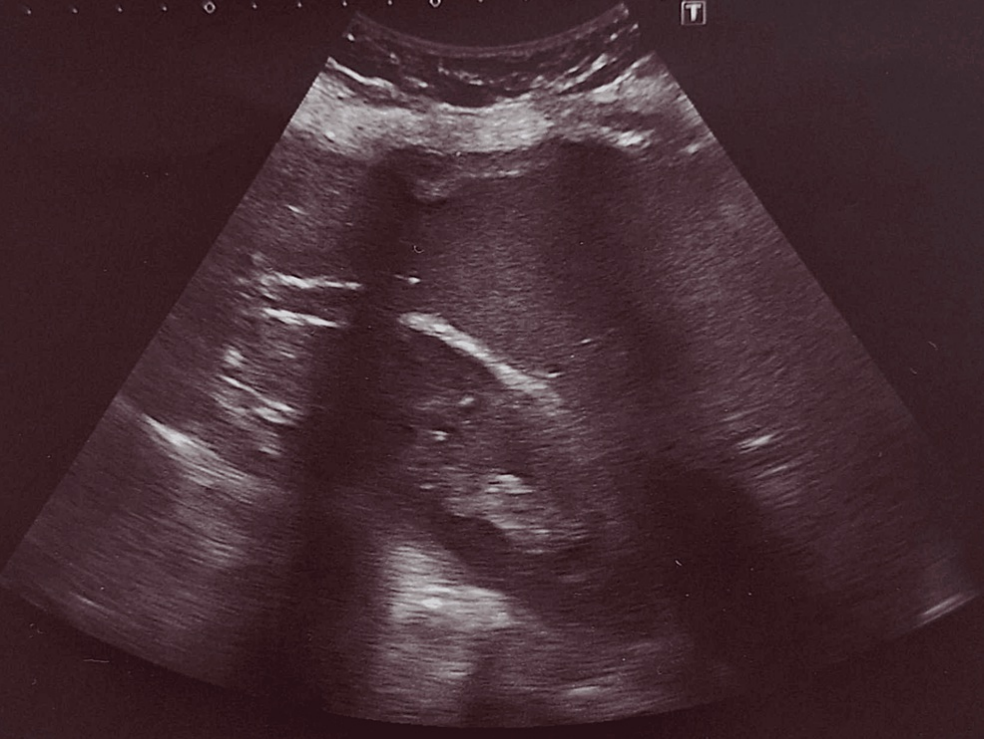
Postoperative ultrasound

There was obstructed drainage with first-degree hydronephrosis but an undilated right ureter. CT-urography showed preserved excretory function of the kidney. There was a narrowing in the pyeloureteral segment. Due to the suspicion of an echinococcal cyst, an ELISA-IgG was performed with an antibody titer of 2.65 mg/dl (positive values >1.1). After pre-operative chemoprophylaxis for five days with albendazole orally 15 mg/kg daily, the cyst was removed by open pericystectomy (surgery-related complications were not observed) and pre-wrapped with gauze with a scolicidal solution. The intervention ended with preservation of the kidney. Vital scolices from the cyst were observed microscopically by the Nakanishi method. The child was consulted at the University Infectious Diseases Hospital for post-operative chemoprophylaxis with albendazole orally 15 mg/kg daily for 30 days. After one year, three of the prophylactic examinations mandatory for Bulgaria were carried out, and echography showed no evidence of recurrence, and the serological test showed a negative antibody titer (Figure 2).

## Discussion

In a synanthropic environment, the main intermediate hosts of hydatid disease are herbivores, mainly sheep. Humans are accidental intermediate hosts and infection usually occurs early in the individual's development, but cyst growth is slow and clinical manifestation is years later; the more common option is that the cyst is discovered incidentally, by imaging on another occasion [2,3]. US examination is the most common method, with good efficiency, but in certain cases, when a more detailed examination of anatomical changes is needed, CT or magnetic resonance is used [2,4]. Urogenital hydatidosis is relatively rare, especially isolated kidney involvement. Epidemiological history taking is an important part of the diagnostic process, where this disease has typical characteristics such as livestock breeding and the presence of dogs, but in our case, the patient does not live in such an area [5]. Symptoms such as dull or sharp lumbar or flank pain, dysuria, hematuria, and inflammatory processes appear extremely rarely. Very rarely, the cyst ruptures in the collecting part of the urogenital system, hydaturia is observed, and scolexis and larvae are released in the urine [5,6].

Diagnosis is difficult, especially in non-endemic areas, but should always be considered in round, double-walled cystic masses of regular shape. Usually, the shape distinguishes them from tumor formations, which are in the first place in the differential-diagnostic plan [7]. Although a tissue parasitosis, moderate eosinophilia is relatively rare (20-50%) in hydatid disease. In case of doubt, it is mandatory to do a serological test, but unfortunately, this method is also not sensitive enough. Healthy cysts tend not to allow antibody formation and the test may be negative. About 20% of all with hydatid disease are seronegative, especially if the cyst is extrahepatic and solitary [8]. In our case, the child presented with a clinical picture resembling pyelonephritis, but the symptoms were mainly due to the growth of the cyst, the compression of adjacent organs, and non-specific inflammatory manifestations. The GP ignored the telltale sign of erythrocyturia, and pain, which are rare symptoms for a common urinary tract infection. A sterile urine sample before the start of antimicrobial therapy, as well as an imaging study, was omitted. Abdominal US would guide the clinician most quickly and accurately.

The treatment of the hydatid cyst is combined, medicinal, percutaneous, or surgical, choosing the most sparing therapy. Our studies show that cysts up to 5cm could be treated only with an antiparasitic medication [9], for larger cysts, invasive treatment is also necessary, and postoperative chemoprophylaxis is mandatory, most often with albendazole 15mg/kg daily, for 1 up to 3 months. Hydatid cysts can recur months and years after removal. Monitoring and prevention are individual, usually for years. In our patient, at the end of the first year, no recurrences were observed.

## Conclusions

Renal hydatid cysts are a rare disease and difficult to diagnose in clinical practice. They must also be considered in non-endemic areas, even if the clinical presentation points to seemingly banal urinary tract infection. Diagnostics are guided by imaging studies; they are especially necessary for hematuria and lingering local pain. Most often, the cyst is detected by abdominal US, after which additional diagnostic and therapeutic behavior is discussed, as well as monitoring, for prevention of recurrences.

## References

[1] Wen H, Vuitton L, Tuxun T, Li J, Vuitton DA, Zhang W, McManus DP (2019). Echinococcosis: advances in the 21st century. Clin Microbiol Rev.

[2] Eckert J, Deplazes P (2004). Biological, epidemiological, and clinical aspects of echinococcosis, a zoonosis of increasing concern. Clin Microbiol Rev.

[3] Lewall DB (1998). Hydatid disease: biology, pathology, imaging and classification. Clin Radiol.

[4] (2003). International classification of ultrasound images in cystic echinococcosis for application in clinical and field epidemiological settings. Acta Trop.

[5] Mladenov B, Dorosiev E (2021). Primary renal echinococcosis - a rare location of hydatid disease. Folia Med (Plovdiv).

[6] Hailu SS, Gebremariam M, Nigussie T, Girma K, Misgea A, Arega G (2023). Isolated renal hydatid cyst in a ten-year-old female child: a rare case report. Urol Case Rep.

[7] Paramythiotis D, Bangeas P, Kofina K, Papadopoulos V, Michalopoulos A (2016). Presence of an isolated hydatid cyst in the left kidney: report of a case of this rare condition managed surgically. Case Rep Urol.

[8] Rexiati M, Mutalifu A, Azhati B, Wang W, Yang H, Sheyhedin I, Wang Y (2014). Diagnosis and surgical treatment of renal hydatid disease: a retrospective analysis of 30 cases. PLoS One.

[9] Todorov T, Vutova K, Donev S, Ivanov A, Katzarov K, Takov D (2005). The types and timing of the degenerative changes seen in the cysts during and after benzimidazole treatment of cystic echinococcosis. Ann Trop Med Parasitol.

